# Poly-substance use and sexual risk behaviours: a cross-sectional comparison of adolescents in mainstream and alternative education settings

**DOI:** 10.1186/s12889-019-6892-0

**Published:** 2019-05-14

**Authors:** Marion Henderson, Catherine Nixon, Martin J. McKee, Denise Smith, Daniel Wight, Lawrie Elliott

**Affiliations:** 1 MRC/CSO Social and Public Health Sciences Unit, University of Glasgow, 200 Renfield Street, Scotland, G2 3AX UK; 2 Department of Nursing and Community Health, Glasgow Caledonian University, Cowcaddens Road, Glasgow, Scotland, G4 OBA UK

**Keywords:** Alternative education settings, Schools, Sexual health, Substance use, Parenting, Health promotion, Young people/adolescents

## Abstract

**Background:**

Surveys of young people under-represent those in alternative education settings (AES), potentially disguising health inequalities. We present the first quantitative UK evidence of health inequalities between AES and mainstream education school (MES) pupils, assessing whether observed inequalities are attributable to socioeconomic, familial, educational and peer factors.

**Methods:**

Cross-sectional, self-reported data on individual- and poly-substance use (PSU: combined tobacco, alcohol and cannabis use) and sexual risk-taking from 219 pupils in AES (mean age 15.9 years) were compared with data from 4024 pupils in MES (mean age 15.5 years). Data were collected from 2008 to 2009 as part of the quasi-experimental evaluation of Healthy Respect 2 (HR2).

**Results:**

AES pupils reported higher levels of substance use, including tobacco use, weekly drunkenness, using cannabis at least once a week and engaging in PSU at least once a week. AES pupils also reported higher levels of sexual health risk behaviours than their MES counterparts, including: earlier sexual activity; less protection against sexually transmitted infections (STIs); and having 3+ lifetime sexual partners. In multivariate analyses, inequalities in sexual risk-taking were fully explained after adjusting for higher deprivation, lower parental monitoring, lower parent-child connectedness, school disengagement and heightened intentions towards early parenthood among AES vs MES pupils. However, an increased risk (OR = 1.73, 95% CI 1.15, 2.60) of weekly PSU was found for AES vs MES pupils after adjusting for these factors and the influence of peer behaviours.

**Conclusion:**

AES pupils are more likely to engage in health risk behaviours, including PSU and sexual risk-taking, compared with MES pupils. AES pupils are a vulnerable group who may not be easily targeted by conventional population-level public health programmes. Health promotion interventions need to be tailored and contextualised for AES pupils, in particular for sexual health and PSU. These could be included within interventions designed to promote broader outcomes such as mental wellbeing, educational engagement, raise future aspirations and promote resilience.

## Background

Although health inequalities among adolescents have been reported to be smaller than those observed for younger or older age groups [1, 2], it is known that health inequalities among adolescents are widening [3]. Health risk behaviours that are adopted during adolescence are known to track strongly into adulthood [4–6]. These behaviours, which include smoking, heavy alcohol consumption and drug use, have all been shown to contribute to excess morbidity and mortality during adulthood [7]. Furthermore, it has been identified that tobacco, alcohol and drug use [8, 9] and sexual risk taking behaviour [9, 10] are two of the major causes of death and illness among adolescents and young adults. It is thus important to understand trends in health inequalities and the underlying social determinants of these in order to develop interventions and policies to reduce the effects of these upon population health [11, 12].

In order to understand factors exacerbating ill-health and perpetuating inequalities, calls have been made for more research focused upon the experience of vulnerable and socially excluded populations [13]. One population that is currently under-represented within the UK literature is adolescents attending Alternative Education Settings (AES). This is because most large scale population surveys of adolescent health in the UK are conducted in mainstream schools, thus excluding AES pupils [14]. AES are defined by the UK Department of Health as education arranged by local authorities for “permanently excluded pupils” and “other children who, because of exclusion, illness or other reasons”, for instance behavioural problems, learning disabilities and developmental delays, “would not receive suitable education”. AES are also attended by pupils “subject to a fixed-period exclusion of more than five days” [15]. The most common reasons for referral to AES in the UK are: physical assault of teaching staff and pupils; persistently disruptive behaviour in the classroom; risk taking behaviours such as youth offending, substance use and high risk sexual behaviour; and teenage pregnancy [16–18]. Around 6500–7000 pupils in Scotland attend these settings each year, with published statistics aggregated to include both primary and secondary schools pupils [19].

Young people in AES, particularly those who are attending due to behavioural problems, disengagement from education and repeat exclusions from mainstream education settings (MES), may be particularly susceptible to adopting health risk behaviours. This is because health risk behaviours such as sexual risk-taking and substance use, which often start during adolescence, are associated with deprivation and social exclusion [20–24]. For example, in a representative sample of 15–16 year olds in Scotland, higher sexual risk was associated with deprivation, living in a lone-parent household, low levels of parental monitoring, living in a care/foster home and having limited educational/career aspirations [25, 26]. These factors are also associated with school exclusion [27], which in turn can increase the likelihood of antisocial and risky behaviours [28]. Where this includes being exposed to and using substances, evidence from adolescent cohort studies suggests that this is associated with poorer sexual health outcomes for adolescents, including earlier sex, higher numbers of sexual partners, earlier pregnancies and risk of contracting sexually transmitted infections (STIs) [29–32].

Although little is known about the health risk behaviours of AES in the UK, evidence utilising cross-sectional, self-reported data from young people in the USA, the Netherlands and New Zealand reveal high rates of health risk behaviours among AES pupils, including cigarette smoking [33–36], alcohol consumption [33–35], drug use [33–37] and sexual risk behaviour (e.g. early sexual initiation, poor contraceptive use and increased levels of teenage pregnancy) [35, 38–42]. The most rigorously conducted of these, the 1998 National Alternative High School Youth Risk Behaviour Survey (ALT-YRBS), used probability sampling techniques to create a nationally representative sample of pupils attending AES (mean age 16.8 years) in the United States [33]. The authors reported that: 64.1% of AES pupils smoked cigarettes; 64.5% consumed alcohol; 49.8% engaged in episodic heavy drinking (i.e. they had consumed 5+ drinks on two or more days in the preceding month); 84.5% had tried marijuana; and 53% reported being current users of marijuana. In all cases, males were significantly more likely than females to use substances [33]. Looking specifically at sexual behaviour, the ALT-YRBS study reported that: 87.8% of AES pupils were sexually active; 22% had been under the age of 13 at sexual initiation; and 50.4% had had four or more sexual partners. Around half (45.6%) of male AES and two thirds (63.9%) of female AES reported that they had not used condoms at last sexual intercourse [33]. Similar prevalence rates were reported in a New Zealand study of AES pupils [43].

Few studies have compared the health risk behaviours of AES and MES pupils [34, 36, 44]. Comparing the prevalence of health risk behaviours found within the 1998 ALT-YRSB with an age- and ethnicity- matched sample from the 1997 Youth Risk Behaviour Survey (YRBS), Grunbaum et al. [34] concluded that AES pupils were significantly more likely than MES pupils to smoke cigarettes (70.1% vs. 36.3%), consume alcohol (64.9% vs. 50.8%), use marijuana (53.9% vs. 26.2%), and be sexually active (85.5% vs. 48.3%). Sexually active AES pupils were also significantly more likely to report that they had had 4+ sexual partners (48.0% vs. 16%) and that they had not used a condom at last sexual intercourse (52.5% vs. 43.1%). The same pattern of significantly elevated risk has been reported in comparative studies of AES and MES pupils conducted in New Zealand [36] and Hong Kong [44].

The high levels of risk taking behaviours observed among AES pupils has resulted in their being described by society as ‘marginalised’, ‘disenfranchised’, ‘troubled’, ‘problem’, ‘bad’ and ‘failing’ youth [45–50]. These descriptions not only stigmatise young people in AES, but act as a barrier to understanding the wider socio-environmental and cultural factors that may perpetuate health inequalities by placing the onus for poor health outcomes upon individual choices and behaviours [50]. However, both quantitative and qualitative evidence suggests that the socioeconomic and familial characteristics of pupils within AES may increase their propensity to display increased levels of health risk-taking behaviours. Young people attending AES disproportionately come from low-income and lone-parent households. They are also more likely to have been exposed to social and familial adversity (e.g. parental mental ill-health, parental substance use and domestic violence), been maltreated as a child and be known to social work and criminal justice systems [16, 51–54]. These same factors have been identified by the UK Government as key drivers of social exclusion, and can increase the health risks of young people over time in the UK and beyond [55–58]. Thus, it is likely that in addition to familial factors, school exclusion contributes to health inequalities for AES pupils by acting as a barrier to young people being exposed to prosocial peers and adults, resulting in them receiving less monitoring and supervision, spending more time with other disadvantaged pupils, and being at increased risk of exposure to risky behaviours, including sexual risk-taking behaviours [59].

Although the literature suggests that disadvantage across multiple social contexts may underpin the increased levels of health risk behaviour observed among AES, to date, studies exploring the antecedents of substance use and sexual risk taking behaviours among AES have focussed mainly upon exploring associations between interpersonal factors and health risk behaviours. Looking first at family factors, it has been reported that AES pupils who perceive greater family connectedness or caring have lower levels of marijuana and cocaine use [60], are less likely to be sexually active [40] and are more likely to use condoms during intercourse [40]. Beyond familial factors, several studies have identified the associations between peers, educational aspirations and substance use. For instance, alcohol and drug use among peers has been found to be associated with substance use among AES, and also contributed significantly to the experience of negative consequences associated with substance use in adulthood [61]. Social isolation (e.g. having fewer friends) was associated with an increase in the number of monthly substances used among AES [62], while having lower educational aspirations was associated with increased cocaine use [60].

While these findings provide valuable insight into the factors associated with elevated levels of risk-taking among AES they are limited by the fact that they focus solely upon explaining how single risk factors, for instance lack of parent-child connectedness, explain the increased levels of risk observed among samples of AES pupils. Within the public health literature it has been identified that the most effective behaviour change interventions are based upon understanding behaviours and the settings in which they occur [63]. The social ecological model (SEM) is a theoretical framework that can be used to demonstrate that human health is shaped through the complex interaction of individual- (e.g. sex, economic status, knowledge, attitudes and behaviour), interpersonal- (e.g. social networks, social and familial support), organisational- (e.g. polices within organisations that affect how services are delivered to individuals), community- (e.g. relationships between organisations and institutions) and policy- (e.g. local and national laws) factors [64]. In order to reduce risk taking among vulnerable and socially excluded groups such as AES pupils, the framework of the SEM can be used to help understand how factors operating at multiple levels contribute to health risk behaviours [65].

Drawing upon the SEM, this study aims to build upon existing research by using multivariable regression models to explore, for the first time, how individual (socioeconomic status, age and gender) and interpersonal (familial, educational and peer) factors contribute to substance use behaviours and sexual risk taking among AES and MES pupils. We also aim to explore whether differences in the underlying socio-economic, familial, educational and peer factors of AES and MES pupils can help to explain why AES have significantly higher rates of health risk behaviours when compared with their contemporaries in MES [50]. Other studies have not compared AES and MES across school settings in this way. In order to meet these aims, three research questions (RQ) will be addressed:

1. Do socio-economic, family, education and peer background factors differ between AES and MES pupils in the UK?
2. Do rates of PSU and sexual risk behaviours differ between AES and MES pupils in the UK?
3. Can potential differences in rates of PSU and sexual risk-taking between AES and MES pupils in the UK be explained by individual (sex, age and socioeconomic circumstances) and interpersonal (family, education, and peer) factors?

In answering these questions, we provide the first UK based estimates of substance use and sexual risk taking behaviour among AES pupils.

## Methods

### Study design

Self-reported sexual risk, PSU and a range of socio-economic, family, education and peer background factors were compared between AES and MES pupil sub-samples of the Healthy Respect 2 (HR2) study; a quasi-experimental evaluation assessing whether the combined provision of sex education with youth-friendly sexual health services, media campaigns and branding improved sexual health knowledge and reduced sexual risk taking among adolescents. Results of the evaluation are reported elsewhere [66, 67].

#### Sub-sample 1 – MES

Cross-sectional, self-reported data were collected from Scottish secondary year 4 (the final year of statutory schooling) pupils aged 14–17 years (mean age 15.5 years) attending 12 mainstream high schools that were participating in a quasi-experimental evaluation of HR2. The intervention area and the schools within it were pre-selected by the HR2 intervention team, and teachers received training in how to deliver a theoretically based sex education programme (SHARE) [68]. Six of the potential 26 secondary schools within the intervention area were approached to participate; the schools we did not approach had participated in a previous implementation and evaluation of SHARE [69]. All of the schools approached agreed to participate. The comparison areas were selected based upon their 1) having comparable teenage pregnancy and termination rates and 2) not using SHARE within the sexual health and relationships curriculum. The educational authority initially invited to participate as our comparison area declined on the basis of not having the capacity to participate in research due to a restructure of services within the area; however, our second choice of educational authorities agreed to participate. As deprivation is a known indicator of early sexual debut and teenage pregnancy [70], comparison area schools were matched with intervention schools according to the percentage of pupils eligible for free school meals (a means-tested indicator of pupil deprivation in the UK). Ten schools with matched demographics were invited to participate, with 6 agreeing to do so.

Data were collected during the academic years 2006/7, 2007/8 and 2008/9 by trained researchers. 5283 (80%) of the 6608 registered pupils completed questionnaires under examination conditions without teachers present, with pupils offered additional help and support if they encountered difficulties. The 20% of pupils excluded from analysis consisted of those with missing outcome data or spoiled questionnaires (5%) and those absent from school due to illness, temporary exclusion or release to attend college or vocational training (15%). Rates of questionnaire exclusion and absenteeism were comparable across data collection years and schools. Postal questionnaires and pre-paid envelopes (*n* = 1025) were left with teachers for pupils who were absent from school; however, less than 1% (*n* = 60) of these were returned. In this paper, data are reported from the 4024 pupils who participated in data collection during the 2007/2008 and 2008/09 sweeps in order to match with the timing of data collection for AES.

#### Sub-sample 2 – AES

Cross-sectional, self-reported data were collected from pupils aged 14–17 years (mean age 15.9 years) attending AES using a shortened, simplified version of the MES questionnaire [66, 67]. The AES and MES were located in the same geographical areas. A total of 17 AES were identified through official lists of local authorities, including special schools, residential schools and special education units attached to mainstream schools; 12 AES agreed to take part. Reasons for non-participation included schools having limited capacity to participate in research and concerns from teachers that the research topic was ‘too sensitive’ for students. Table 1 shows that the schools which participated provided education for young people with: physical disabilities (*n* = 1), moderate/complex special education needs (SEN) (*n* = 4), socio-emotional and behavioural difficulties (SEBD) (*n* = 5); academic difficulties and vocational training (*n* = 5); and young women disengaged from education by pregnancy/early parenthood (*n* = 1). Eight of the schools provided education to a single AES pupil type (e.g. SEN), whilst four schools described themselves as providing care to more than one AES pupil type (e.g. SEN and SEBD).

**Table 1:** AES by special educational needs, age and education support

Alternative Education Settings in the Study				
ID	Location	Pupil Type	Ages^a^	Type of Education
AES1	East of Scotland	Special educational needs	12–16	Individualized education for pupils with Special Educational Needs
AES2	East of Scotland	Requires personal, social and practical skills to move from school to work	15–17	Standard Grades (replaced Scottish ‘O’ Grades), word processing skills, job search skills, workshops and work placements
AES3	East of Scotland	Academic difficulties; literacy issues	12–16	Individualized education, develop personal and social skills
AES4	East of Scotland	Pregnant girls or young mothers	14–17	Standard Grades
AES5	East of Scotland	Social, emotional and behavioural difficulties,	12–16	Individualized education, develop personal and social skills
AES6	East of Scotland	Social, emotional and behavioural difficulties; academic difficulties	14–17	Additional support needs, develop personal and social skills
AES7	East of Scotland	Academic difficulties	16–19	Job search skills, workshops and work placements
AES8	East of Scotland	Social, emotional and behavioural difficulties; academic difficulties	16–19	Additional support needs, develop personal and social skills
AES9	West of Scotland	Physical disabilities; complex learning difficulties	5–18	Additional support needs, develop personal and social skills
AES10	West of Scotland	Social, emotional and behavioural difficulties; moderate learning difficulties	14–18	Vocational skill development
AES11	West of Scotland	Special educational needs	5–19	Individualized education for pupils with Special Educational Needs
AES12	West of Scotland	Social, emotional and behavioural difficulties	12–17	Individualized education, develop personal and social skills

^a^Survey data was collected from pupils aged 14–17 years, these age ranges are included to show the wider demographics of the schools surveyed

Data were collected either under examination conditions without teachers present, with researcher support to answer questions as they arose (85%) or, if problems with literacy or attention prevented this, as a one-to-one semi-structured interview delivered by trained researchers (15%). Where additional support was required, researchers were provided with a list of alternative words and examples that could be used to promote better understanding of questions. Postal questionnaires were not left for absentees as the high level of learning and attention difficulties in this population would have meant that the questionnaires would have had to be completed with teacher support, which would have been inappropriate given the sensitivity of the topics investigated. Of the 233 pupils eligible to participate, 219 (94%) completed a questionnaire, with the remaining 6% consisting of those who did not want to participate or who were identified by AES staff as likely to struggle with content and comprehension.

### Outcome measures

This paper focuses on three primary outcomes associated with PSU and sexual risk-taking: 1) whether young people were poly-substance users (based upon the DSM-IV diagnostic criteria that polysubstance dependence relies on individuals being reliant upon three or more substances [71] we defined PSU as having used alcohol, tobacco and cannabis); and 2) had engaged in penetrative sex (defined as anal or vaginal intercourse); and 3) were protected against both pregnancy and STIs either through engaging in abstinence or opting to use condoms at sexual debut and beyond. The proportion of young people using each of the three substances measured (alcohol, tobacco or cannabis) is reported in descriptive statistics, before being combined into PSU for multivariable models due to the small number of pupils using some of these substances. Secondary outcomes relating to sexual risk behaviour focussed upon: whether sexual debut was planned; age at sexual debut, in particular being under 13 years at first sex; partner age at sexual debut; whether pupils felt pressured at sexual debut; whether sexual debut was under the influence of drugs or alcohol; whether pupils discussed using condoms with their partner at sexual debut; and reported having more than the median number of sexual partners. Except where specified, results are presented separately by gender due to existing evidence suggesting that gender differences exist in the adoption of health risk behaviours in AES and MES [14]. Table 2 provides a summary of the primary and secondary outcomes, the questions asked to determine these outcomes, and how each outcome was coded.

**Table 2:** Description of outcome and explanatory variables

Variable	Questions asked	Variable creation details
Primary Outcomes		
Substance Use and Poly-substance	Pupils in both MES and AES were asked three questions about substances: “how often in the last twelve months have you got drunk/ used cannabis, hash, weed or grass /smoked tobacco (cigarettes)?” Answers were given on 6 point scale for alcohol and cannabis use (1 = never, 6 = more than once a week) and 7 point scale for tobacco use (1 = never, 7 = every day). For each substance, responses dichotomised into used once a week or more vs. less often.	A binary variable was created for each substance by dichotomizing responses into “used once a week or more” vs. “less often”. A binary variable for poly-substance use was created by dichotomizing use of all three substances (alcohol, tobacco and cannabis) into “once a week or more” vs. “less often”. The decision to define poly-substance use as using all three substances was based upon the DSM-IV diagnostic criteria that polysubstance dependence relies on individuals being reliant upon three or more substances [63]
Has had penetrative sex activity	Pupils in both MES and AES were asked “have you ever had penetrative sex? Response options were “yes” and “no”.	A binary variable was created. Pupils with missing data who had provided answers to subsequent questions about the circumstances surrounding were recoded to “yes” and included in the analysis. Otherwise, missing data was excluded from the analysis.
Protected against both pregnancy and STIs, either through abstinence or by using condoms or condoms plus another contraceptive at sexual debut or beyond.	Pupils in both MES and AES were asked two questions about being protected against pregnancies and STIs. 1) “when you first had penetrative sex, did you or your partner use protection against pregnancy or sexually transmitted infections?” Responses included: “No”, “Penis pulled out before ejaculation or cumming”, “condom was used”, “I/my partner was on the pill”, “I/my partner used emergency contraception (the morning after pill)”, “other (please write in)” and “don’t know”. 2) “thinking carefully about all the times you have had penetrative sex ever… how often did you or your partner use a condom?” Responses included: “never”, “not very often”, “about half the time”, “most of the time”, “always” and “don’t know”.	A binary variable was created for protection against pregnancy and STIs, either by always using a condom or through abstinence. Pupils who stated that they had used a condom at sexual debut (question 1) and also reported that they had “always” used a condom when having penetrative sex (question 2) were coded as having always been protected against pregnancy and STIs. As were pupils who had answered “no” to the question “have you ever had penetrative sex?” Pupils with missing data for these questions were excluded from the analysis.
Secondary Outcomes		
Sexual debut was planned	Pupils in both MES and AES were asked which of the following statements best described the circumstances surrounding their sexual debut: “I planned it by myself without my partner”, “we planned it together”, “it was completely unexpected” and “I can’t remember”.	Pupils who answered “I planned it by myself without my partner” or “we planned it together” were coded as having had a planned sexual debut. Those who answered “it just happened on the spur of the moment”, “I expected it to happen soon but was not sure when” or “it was completely unexpected” were coded as having had an unplanned sexual debut. Pupils who answered “I can’t remember” or had missing data were coded as “can’t remember/missing”.
Age at sexual debut	Pupils in both MES and AES were asked “when you first had penetrative sex, how old were you?”.	Responses were recoded as “under 13 years of age” vs. “13 years of age or over” vs “missing”. Age 13 was chosen as in the UK, sexual activity under this age is a reportable offence.
Partner age at sexual debut	Pupils in both MES and AES were asked “how old was the person you had penetrative sex with?” Age differences ranged from −2 years (younger) to 42 years (older).	Responses were recoded as “three plus years older” vs. “1–2 years older, the same age or 1–2 years younger” vs. “can’t remember/missing”.
Felt pressured at sexual debut	Pupils in MES were asked which of the following statements best described the circumstances surrounding their sexual debut: “he/she put a lot of pressure on me”, “he/she put pressure on me”, “he/she put a bit of pressure on me”, “there was no pressure either way”, “I put a bit of pressure on him/her”, “I put pressure on him/her”, “I put a lot of pressure on him/her”. Pupils in AES were provided with a condensed response set of “he/she put pressure on me”, “there was no pressure either way” and “I put pressure on him/her”.	As we were interested in the proportion of pupils who had felt pressured into having sex, were responses were recoded into “I felt pressured at sexual debut” vs. “I did not feel pressured at sexual debut” vs. “missing”. MES pupils who answered “he/she put a lot of pressure on me” and “he/she put a bit of pressure on me” and AES pupils who answered “he/she put pressure on me” were coded as having experienced pressure. All other responses were coded as not having experienced pressure.
Sexual debut was under the influence of drugs or alcohol	Pupils in both MES and AES were asked “were you drunk or stoned when you first had penetrative sex?” Responses included “yes” and “no”.	Responses were recoded as “yes” vs. “no” vs. “missing”.
Discussed using condoms with partner at sexual debut	Pupils in both MES and AES were asked “did you talk about protecting yourself from pregnancy and sexually transmitted infections with the person you had penetrative sex with before having penetrative sex for the first time?” Responses included “yes”, “no” and “can’t remember”	Responses were recoded as “yes” vs. “no” vs. “can’t remember/missing”.
Has had more than the median number of partners	Pupils in both MES and AES were asked “how many people have you ever had penetrative sex with?”	Responses were recoded into “1–2” and “3+” partners, based upon a median response of 2 partners in a positively (right-tailed) skewed distribution. Missing data was coded as a category and included in analyses.
Explanatory variables: pupil characteristics		
Pupil sex	Pupils in both MES and AES were asked “are you a male or a female?”	Responses were left as a binary variable comparing “male” vs. “female”.
Pupil age	Pupils were asked “what month and year were you born?”	Pupil’s age was calculated in years by subtracting the month and year of birth supplied from the month and year questionnaire data was completed. Comparing the mean ages of pupils in AES and MES settings using independent samples t-tests highlighted that AES pupils were significantly older than MES pupils (AES mean age 15.9 vs. MES mean age 15.5; t(214) = 6.017, *p* = 0.000). As the likelihood of engaging in sexual activity increases with age, and there was the potential for a dose related response for secondary sexual health outcomes such as the number of sexual partners, age was dichotomized above and below the mean age of MES pupils to control for any increased levels of risk associated with age.
Explanatory variables: socio-economic circumstances		
Family composition	MES pupils were asked “when you are at home, which adults do you normally stay with?” AES pupils were asked “over the past two years, which adults have you stayed with most of the time?” Possible responses included “mother”, “father”, “step-mother”, “step-father”, “grandmother”, “grandfather”, “I live in a care or foster home” and “other”. Pupils could tick all that applied.	Responses were recoded into “lives with both biological parents”, “lives in a single parent household”, “lives in a reconstituted (step) family” and “lives in a care or foster home”. Missing data was coded as a category and included in the analysis.
Maternal and paternal employment status	Pupils were asked if their mother/father was “in full time paid work”, “in part time paid work”, in “full time housework”, “unemployed”, “a student”, “sick or disabled” or “retired”. Young people could also state that they were “not sure” and that they did not have a “mother/female guardian” or “father/male guardian”.	Responses were recoded to “works full time”, “work part time”, “is unemployed” (e.g. in housework, unemployed, student, sick/disabled or retired) and “no guardian/missing”.
Explanatory variables: family influences		
Frequency of parent-child arguments	Pupils in MES and AES were asked “how often do you have serious disagreements or arguments with your parents/guardians/carers about things, for instance drinking, your friends, homework, tidiness or what you wear?” Responses included “every day”, “most days”, “weekly”, “less often” and “never”.	Responses were recoded into “argues with parents at least once a week” vs. “argues with parents less often”. Missing data was coded as a category and included in the analysis.
Parent-child connectedness	Pupils in MES and AES were asked how strongly they agreed with the following statements using a 5 point scale (1 = strongly agree, 5 = strongly disagree), my parents: “sense when I’m upset about something”, “try to control everything I do”, “encourage me to talk about my difficulties” and “treat me like a baby”.	A mean score (alpha = 0.592) was created for each participant, and recoded into high and low connectedness based upon mean scores above /below the median in a negatively (left-tailed) skewed distribution. Missing data was coded as a category and included in the analysis.
Family time	Pupils in MES and AES were asked about the amount of time they spent “eating a meal”, “going for a walk or playing sport” and “going places” with their parents, and “doing other things as a family group”. Responses were based on a 4 point scale (AES 1 = more than once a week, 4 = never; MES 1 = everyday, 4 = never).	Items on “eating a meal” and “going for walks” together were combined to form a mean score (alpha = 0.509) for each participant. Other items were excluded due to reliability testing showing low internal consistency (alpha < 0.2) when all items were combined into a scale. The combined family time score was then recoded to “frequent family time” vs. “less frequent family time” based upon mean scores above /below the median in a positively (right-tailed) skewed distribution. Missing data was coded as a category and included in the analysis.
Spending money (proxy variable for young people”s autonomy)	Pupils in both MES and AES were asked “each week, how much money do you get to spend on things you want?” using a seven point scale (1 = nothing, 2 = less than £7, 3 = £7–12 increasing through £5 increments to 7 = £30 or more).	Responses were recoded into “less than £25 a week” and “£25 a week or more”. Missing data was coded as a category and included in the analysis.
Parental monitoring	Pupils in both MES and AES were asked two questions about parental monitoring: “do you have to be back by a certain time” and “does anybody stay up until you get home”. Responses were based on a 4 point scale, with 1 = always and 4 = never.	Items on “do you have to be back by a certain time” and “does anybody stay up until you get home” were combined to form a mean score (alpha = 0.591) for each participant. Scores were recoded into high and low parental morning based upon mean scores above/below the median in a positively (right-tailed) skewed distribution. Missing data was coded as a category and included in the analysis.
Explanatory variables: educational factors		
Intended school leaving age (Educational engagement)	Pupils were asked when they intended to leave school. Pupils in MES were asked if they intended to leave school as soon as legally possible (“at the end of S4” or “Christmas S5”), intended to remain in school until completion of upper school exams (“the end of S5” or “the end of S6”). As pupils in AES were likely to be older they were asked if they wanted to leave “at age 16/as soon as possible” or “at an older age”. A “don’t know” category was provided in both questionnaires.	Responses of “at the end of S4 or Christmas S5” among MES pupils and “at age 16/as soon as possible” were recoded as leaving school “as soon as possible”. Response of “the end of S5”, “the end of S6” for MES pupils and “at an older age” were recoded as “remain in school”. Missing answers and “don’t know” were combined as a category and included in the analysis.
Future expectations of: employment, education and training by age 19	Pupils in both AES and MES used a series of 3- (AES, 1 = likely, 3 = unlikely) and 5-point scales (MES, 1 = very likely, 5 = very unlikely) to report the perceived likelihood of their being: “in a secure job/apprenticeship”, “in a training scheme”, “at college or university”, or “unemployed or in casual work” in the next three years.	Responses of “likely” and “very likely” were recoded into “will likely be in education, employment and training” whilst responses of “unlikely” and “very unlikely” were coded as “will not be in education, employment or training”. Missing answers and “unsure” categories were combined and included in the analysis.
Future expectations of: parenthood by age 19	Pupils in both AES and MES used a series of 3 (AES, 1 = likely, 3 = unlikely) and 5-point scales (MES, 1 = very likely, 5 = very unlikely) to report the perceived likelihood of having “a child/children” in the next three years.	Responses of “likely” and “very likely” were recoded into “will likely be a parent” whilst responses of “unlikely” and “very unlikely” were coded as “will not be a parent”. Missing answers and “unsure” categories were combined and included in the analysis.
Explanatory variables: peer influences		
Peer engagement with education	Pupils in both AES and MES were asked what proportion of their peers had “left school”, using a 5 point scale (1 = none, 5 = all).	Responses were recoded into “more than half” vs. “less than half”. Missing data was coded as a category and included in the analysis.
Peer smoking behaviour	Pupils in both AES and MES were asked what proportion of their peers	Responses were recoded into “more than half” vs. “less than half”. Missing data was coded as a category and included in the analysis.
Peer sexual behaviour	Pupils in both AES and MES were asked what proportion of their peers do “you think have had penetrative sex”, using a 5 point scale (1 = none, 5 = all).	Responses were recoded into “more than half” vs. “less than half”. Missing data was coded as a category and included in the analysis.

### Explanatory variables

We explored whether variations in substance use and sexual risk-taking between AES and MES pupils were explained by individual (sex, age and socioeconomic circumstances) and interpersonal (family, education, and peer) factors. To do this we drew upon known predictors of adolescent sexual risk-taking and substance use, including: family composition; maternal and paternal employment status; frequency of parent-child arguments; parent-child connectedness; family time; spending money (a measure of financial autonomy [72]); parental monitoring; intended school leaving age; future expectations of employment, education and training; future expectations of parenthood; and peer behaviours, including peer engagement with education, peer smoking behaviour and peer sexual behaviour [24, 66, 73–75]. Table 2 lists the questions that were linked to each of the four broad explanatory factors and how responses were recoded for analytical purposes. All assessments were based on previously used, validated instruments [76].

### Analysis

Data were analysed using SPSS version 19. Descriptive statistics and binary logistic regression models were used to answer RQ1 and RQ2. To address RQ3, multivariable logistic regression models were constructed for the three primary outcomes: PSU, whether pupils had had penetrative sex; and protection against both pregnancy and STIs. Univariate analyses (e.g. Table 3) were stratified by gender, however, due to the small number (*n* = 219) of AES pupils surveyed, multivariable regression models were not stratified by gender. For multivariable analyses, four regression models were constructed: Model 1 controlled for the effects of individual factors (e.g. sex, age and socioeconomic circumstances), while Models 2–4 adjusted for the interpersonal factors (Model 2 for the additional effects of family influences; Model 3 for the additional effects of education; and Model 4 for the additional effects of peer influences). Multivariable regression models were not constructed for the secondary sexual health outcomes as it was considered that the small numbers of sexually active AES pupils (*n* = 130) would result in there not being sufficient degrees of freedom to allow for meaningful estimates to be produced. Missing data was patterned by setting, with AES pupils more likely to report that they did not known the answer, or have missing data, for demographic and educational variables. To prevent further reduction in sample size for AES and due to small numbers in each category, missing values were combined with “do not know” answers, dummy coded and included as a category within regression analyses.

**Table 3:** Socio-demographic and familial characteristics of AES and MES Pupils

	Female			Male		
	AES	MES	AES vs. MES	AES	MES	AES vs. MES
	% (n/N)	% (n/N)	OR (95% CI)^₮^	% (n/N)	% (n/N)	OR (95% CI)
Family composition						
Lives with both biological parents	41.2 (28/68)	63.5 (1231/1939)	1.00	37.7 (57/151)	63.5 (1323/2085)	1.00
Lives in single parent household	**38.2 (26/68)**	**23.2 (450/1939)**	**2.54 (1.47, 4.38)**	**39.1 (59/151)**	**24.6 (513/2085)**	**2.70 (1.83, 3.90)**
Lives in reconstituted household	5.9 (4/68)	9.3 (180/1939)	0.98 (0.34, 2.82)	8.6 (13/151)	8.5 (171/2085)	1.77 (0.95, 3.29)
Lives in residential or foster care	**10.3 (7/68)**	**2.0 (39/1939)**	**7.89 (3.25, 19.17)**	**11.9 (18/151)**	**1.8 (38/2085)**	**10.99 (5.91, 20.45)**
Not known/Missing	4.4 (3/68)	2.0 (39/1939)	3.38 (0.99, 11.60)	2.6 (4/151)	1.9 (4/2085)	2.32 (0.80, 6.71)
Paternal employment status						
Full time employment	55.9 (38/68)	77.3 (1499/1939)	1.00	51.7 (78/151)	79.5 (1658/2085)	1.00
Part time employment	7.4 (5/68)	4.5 (88/1939)	2.24 (0.86, 5.84)	**5.3 (8/151)**	**3.8 (79/2085)**	**3.63 (2.37, 5.58)**
Unemployed	8.8 (6/68)	7.0 (136/1939)	1.74 (0.72, 4.19)	**20.5 (31/151)**	**7.1 (149/2085)**	**4.42 (2.82, 6.93)**
No male guardian/missing	**27.9 (1968)**	**11.1 (216/1939)**	**3.47 (1.97, 6.13)**	**22.5 (34/151)**	**9.5 (199/2085)**	**2.15 (1.01, 4.61)**
Maternal employment status						
Full time employment	22.1 (15/68)	47.8 (926/1939)	1.00	31.1 (47/151)	52.2 (1088/2085)	1.00
Part time employment	17.6 (12/68)	25.9 (502/1939)	1.48 (0.68, 3.18)	11.9 (18/151)	22.5 (469/2085)	0.89 (0.51, 1.55)
Unemployed	**45.6 (31/68)**	**14.6 (284/1939)**	**6.74 (3.59, 12.66)**	**38.4 (58/151)**	**14.0 (291/2085)**	**4.64 (3.08, 6.92)**
No female guardian/missing	**14.7 (10/68)**	**11.7 (227/1939)**	**2.72 (1.21, 6.13)**	**18.5 (28/151)**	**11.4 (237/2085)**	**2.74 (1.68, 4.46)**
Frequency of parent-child arguments						
At least once a week	48.5 (33/68)	53.1 (1029/1939)	1.00	37.7 (57/151)	46.8 (975/2085)	1.00
Less than once a week	47.1 (32/68)	46.1 (894/1939)	1.12 (0.68, 1.83)	56.3 (85/151)	52.2 (1088/2085)	1.34 (0.95, 1.89)
Not known/missing	**4.4 (3/68)**	**0.8 (16/1939)**	**5.85 (1.62, 21.05)**	**6.0 (9/151)**	**1.1 (22/2085)**	**7.00 (3.08, 15.89)**
Parent-child connectedness						
High levels of reported connectedness	47.1 (32/68)	57.8 (1121/1939)	1.00	49.7 (75/151)	54.7 (1140/2085)	1.00
Low levels of reported connectedness	45.6 (31/68)	41.6 (806/1939)	1.35 (0.82, 2.23)	44.4 (67/151)	44.3 (924/2085)	1.10 (0.78, 1.55)
Not known/missing	**7.4 (5/68)**	**0.6 (12/1939)**	**14.60 (4.86, 43.89)**	**6.0 (9/151)**	**1.0 (21/2085)**	**6.51 (2.88, 14.72)**
Family time						
Frequently eats meals and go places as a family	32.4 (22/68)	51.1 (991/1939)	1.00	31.1 (47/151)	50.6 (1056/2085)	1.00
Infrequently eats meals and go places as a family	**61.8 (42/68)**	**48.4 (939/1939)**	**2.02 (1.19, 3.40)**	**63.6 (96/151)**	**48.5 (1012/2085)**	**2.13 (1.49, 3.05)**
Not known/missing	**5.9 (4/68)**	**0.5 (9/1939)**	**20.02 (5.73, 70.00)**	**5.3 (8/151)**	**0.8 (17/2085)**	**10.57 (4.34, 25.74)**
Spending money						
£24 or less per week	45.6 (31/68)	20.1 (389/1939)	1.00	51.0 (77/151)	22.5 (470/2085)	1.00
£25 or more per week	**44.1 (30/68)**	**77.8 (1509/1939)**	**4.01 (2.40, 6.70)**	**46.4 (70/151)**	**74.6 (1556/2085)**	**3.64 (2.59, 5.12)**
Not known/missing	**10.3 (7/68)**	**2.1 (41/1939)**	**8.59 (3.57, 20.69)**	2.6 (4/151)	2.8 (59/2085)	1.51 (0.53, 4.27)
Parental monitoring						
High levels of parental monitoring	41.2 (28/68)	64.5 (1121/1939)	1.00	38.4 (58/151)	50.2(1047/2085)	1.00
Low levels of parental monitoring	**57.4 (39/68)**	**34.5 (806/1939)**	**2.60 (1.59, 4.27)**	**58.3 (88/151)**	**48.2 (1006/2085)**	**1.58 (1.12, 2.22)**
Not known/missing	1.5 (1/68)	1.0 (12/1939)	2.23 (0.39, 17.22)	3.3 (5/151)	1.5 (32/2085)	2.20 (0.84, 5.72)
Intended school leaving age						
Remain at school beyond legal leaving age	26.5 (18/68)	71.2 (1381/1939)	1.00	13.2 (20/151)	63.2 (1318/2085)	1.00
Leave school as soon as legally possible	**55.9 (38/68)**	**17.7 (344/1939)**	**8.48 (4.79, 15.03)**	**63.6 (96/151)**	**23.0 (480/2085)**	**13.18 (8.05, 21.58)**
Not known/missing	**17.6 (12/68)**	**11.0 (214/1939)**	**4.30 (2.04, 9.06)**	**23.2 (35/151)**	**13.8 (287/2085)**	**8.04 (4.57, 14.13)**
Expectations of employment, education and training						
Will be in education, employment or training	82.4 (56/68)	92.7 (1797/1939)	1.00	75.5 (114/151)	91.6 (1909/2085)	1.00
Will not be in education, employment or training	**7.4 (5/68)**	**3.0 (59/1939)**	**2.71 (1.20, 6.12)**	**13.2 (20/151)**	**2.3 (48/2085)**	**6.98 (4.01, 12.15)**
Not known/missing	**10.3 (7/68)**	**4.3 (83/1939)**	**2.71 (1.05, 7.04)**	**11.3 (17/151)**	**6.1 (128/2085)**	**2.22 (1.30, 3.82)**
Expectations of parenthood						
Will not be a parent	42.6 (29/68)	79.6 (1544/1939)	1.00	29.1 (44/151)	71.2 (1485//2085)	1.00
Will be a parent	**22.1 (15/68)**	**4.2 (82/1939)**	**6.41 (3.47, 11.85)**	**24.5 (37/ 151)**	**7.2 (151/ /2085)**	**4.16 (2.77, 6.24)**
Not known/missing	**35.3 (24/68)**	**16.1 (313/1939)**	**2.83 (1.70, 4.73)**	**46.4 (70/151)**	**21.5 (449//2085)**	**3.15 (2.25, 4.41)**
Peer behaviours						
More than half of peers have left school	41.2 (28/68)	4.5 (87/1939)	1.00	35.1 (53/151)	4.1 (83/2085)	1.00
Less than half of peers have left school	**51.5 (35/68)**	**93.5 (1813/1939)**	**0.15 (0.10, 0.20)**	**56.3 (85/151)**	**93.6 (1951/2085)**	**0.17 (0.09, 0.33)**
Not known/missing	7.4 (5/68)	2.0 (39/1939)	2.51 (0.90, 6.99)	**8.6 (13/151)**	**2.4 (51/2085)**	**2.51 (1.24, 5.04)**
More than half of peers smoke tobacco	52.9 (36/68)	14.2 (1640/1939)	1.00	42.4 (64/151)	11.2 (234/2085)	1.00
Less than half of peers smoke tobacco	**39.7 (27/68)**	**84.6 (276/1939)**	**0.08 (0.03, 0.21)**	**49.0 (74/151)**	**86.9 (1812/2085)**	**0.12 (0.06, 0.24)**
Not known/missing	7.4 (5/68)	1.2 (23/1939)	0.60 (0.22, 1.68)	8.6 (13/151)	1.9 (39/2085)	0.82 (0.41, 1.63)
More than half of peers have had sex	45.6 (31/68)	25.9 (502/1939)	1.00	45.0 (68/151)	22.0 (459/2085)	1.00
Less than half of peers have had sex	**42.6 (29/68)**	**72.1 (1398/1939)**	**0.29 (0.18, 0.47)**	**47.0 (71/151)**	**75.8 (1580/2085)**	**0.28 (0.20, 0.40)**
Not known/missing	**11.8 (8/68)**	**2.0 (39/1939)**	**0.30 (0.13, 0.70)**	7.9 (12/151)	2.2 (46/2085)	0.57 (0.29, 1.13)

Results significant at *p*<0.05 are in bold

## Results

### RQ1 do socio-economic, family, education and peer background factors differ between AES and MES pupils in the UK?

Table 3 shows that compared with MES pupils, AES pupils were more likely to be living in lone parent families or in residential/foster care placements, and have non-employed parents. Looking at family factors, no difference was observed in the proportions of AES and MES pupils reporting frequent arguments with their parents and experiencing low levels of connectedness to their parents or guardians; however AES pupils were more likely to report that they had limited family time and had higher levels of financial autonomy (measured using spending money). AES pupils also reported significantly lower levels of parental monitoring. AES pupils intended to leave school as soon as it was legally possible, had lower expectations of being in education, employment or training in the next three years, and were more likely to anticipate that they would become parents. AES pupils were also more likely to report that they had peers who engaged in health risk behaviours. Broadly, the same patterning of socio-economic, family, education and peer factors was observed for male and female pupils.

### RQ2: do rates of PSU and sexual risk behaviours differ between AES and MES pupils in the UK?

Tables 4 and 5 report the results of the regression analyses comparing health risk behaviours for AES and MES pupils. Compared with MES pupils, Table 4 shows that AES pupils had increased odds of weekly tobacco use (Male OR = 9.67, 95% CI 6.73, 13.90; Female OR = 5.14, 95% CI 3.10, 8.54), weekly drunkenness (Male OR = 2.79, 95% CI 1.85, 4.21; Female OR = 2.02, 95% CI 1.12, 3.65), using cannabis at least once a week (Male OR = 7.15, 95% CI 4.74, 10.78; Female OR = 9.34, 95% CI 4.25, 20.54) and engaging in PSU at least once a week (Male OR = 4.35, 95% CI 3.03, 5.88; Female OR = 3.70, 95% CI 2.17, 6.25). Looking at sexual behaviour, Table 5 shows that compared with MES pupils, AES pupils had increased odds of having had penetrative sex (Male OR = 3.22, 95% CI 2.29, 4.51, Female OR = 2.36, 95% CI 1.45, 3.85) and being exposed to both pregnancy and STIs by not engaging in abstinence or failing to use condoms at sexual debut and beyond (Male OR = 3.90, 95% CI 2.63, 5.79, Female OR = 2.01, 95% CI 1.12, 3.60).

**Table 4:** Substance use amongst AES and MES pupils

	Female			Male		
	AES	MES	AES vs. MES	AES	MES	AES vs. MES
	% (n/N)	% (n/N)	OR (95% CI)	% (n/N)	% (n/N)	OR (95% CI)
Tobacco use						
Has never smoked tobacco	42.6 (29/68)	69.1 (1338/1939)	1.00	34.4 (52/151)	73.9 (1541/2085)	1.00
Smokes tobacco less than once a week	2.9 (2/68)	14.1 (274/1939)	0.34 (0.08, 1.42)	4.0 (6/151)	11.6 (242/2085)	0.74 (0.31, 1.73)
Smokes tobacco at least once a week	**51.5 (35/68)**	**16.2 (314/1939)**	**5.14 (3.10, 8.54)**	**60.9 (92/151)**	**14 (282/2085)**	**9.67 (6.73, 13.90)**
Not known/missing	**2.9 (2/68)**	**0.7 (13/1939)**	**7.10 (1.53, 32.90)**	0.7 (1/151)	1.0 (20/2085)	1.48 (0.20, 11.25)
Alcohol use						
Has never drank alcohol to the point of drunkenness	29.4 (20/68)	29.9 (580/1939)	1.00	27.8 (42/151)	33.7 (702/2085)	1.00
Drinks alcohol to the point of drunkenness less than once a week	27.9 (19/68)	49.1 (953/1939)	0.58 (0.31, 1.09)	29.1 (44/151)	47.2 (985/2085)	0.75 (0.48, 1.15)
Drinks alcohol to the point of drunkenness at least once a week	**39.7 (27/68)**	**20.0 (388/1939)**	**2.02 (1.12, 3.65)**	**41.7 (63/151)**	**18.1 (377/2085)**	**2.79 (1.85, 4.21)**
Not known/missing	2.9 (2/68)	0.9 (18/1939)	3.22 (0.70, 14.82)	1.3 (2/151)	1.0 (21/2085)	1.59 (0.36, 7.02)
Cannabis use						
Has never smoked cannabis	57.4 (39/68)	85.6 (1659/1939)	1.00	50.3 (76/151)	79.4 (1655/2085)	1.00
Smokes cannabis less than once a week	**23.5 (16/68)**	**11.7 (227/1939)**	**3.00 (1.65, 5.45)**	**16.6 (25/151)**	**13.2 (275/2085)**	**1.98 (1.24, 3.17)**
Smokes cannabis at least once a week	**13.2 (9/68)**	**2.1 (41/1939)**	**9.34 (4.25, 20.54)**	**29.1 (44/151)**	**6.4 (134/2085)**	**7.15 (4.74, 10.78)**
Not known/missing	**5.9 (4/68)**	**0.6 (12/1939)**	**14.18 (4.38, 45.93)**	**4.0 (6/151)**	**1.0 (21/2085)**	**6.22 (2.44, 15.86)**
Poly-substance use						
Does not use all three substances at least once a week	64.7 (44/68)	87.6 (1698/1939)	1.00	58.3 (88/151)	85.3 (1779/2085)	1.00
Uses all three substances at least once a week	**32.4 (22/68)**	**12.0 (232/1939)**	**3.70 (2.17, 6.25)**	**41.1 (62/151)**	**14.0 (292/2085)**	**4.35 (3.03, 5.88)**
Not known/missing	2.90 (2/68)	0.40 (8/1939)	0.38 (0.07, 1.88)	0.70 (1/151)	0.70 (14/2085)	2.94 (0.38, 25.00)

Results significant at *p*<0.05 are in bold

**Table 5:** Sexual risk taking amongst AES and MES pupils

	Female			Male			
	AES	MES	AES vs. MES	AES		MES	AES vs. MES
	% (n/N)	% (n/N)	OR (95% CI)^₮^	% (n/N)		% (n/N)	OR (95% CI)^₮^
Has had penetrative sex							
No	35.3 (24/68)	53.1 (1029/1939)	1.00	28.5 (43/151)		54.0 (1126//2085)	1.00
Yes	**57.4 (39/68)**	**36.3 (704/1939)**	**2.36 (1.45, 3.85)**	**60.3 (91/151)**		**32.0 (668//2085)**	**3.22 (2.29, 4.51)**
Not known/missing	7.4 (5/68)	10.6 (206/1939)	0.67 (0.27, 1.68)	11.3 (17/151)		14.0 (291//2085)	0.78 (0.47, 1.32)
Protected against both pregnancy and STIs, either through abstinence or by using condoms or condoms plus another contraceptive at sexual debut or beyond							
Yes	67.6 (46/68)	76.2 (1477/1939)	1.00	52.3 (79/151)		75.3 (1570//2085)	1.00
No	**23.5 (16/68)**	**13.2 (256/1939)**	**2.01 (1.12, 3.60)**	**29.1 (44/151)**		**10.7 (224//2085)**	**3.90 (2.63, 5.79)**
Not known/missing	8.8 (6/68)	10.6 (206/1939)	0.94 (0.40, 2.22)	18.5 (28/151)		14.0 (291//2085)	1.91 (1.22, 3.00)
Sexual debut was planned							
Yes	12.8 (5/39)	20.9 (147/704)	1.00	13.0 (14.3/91/)		22.2 (148/668)	1.00
No	76.9 (30/39)	63.9 (450/704)	1.96 (0.75, 5.14)	70.3	(64/91/)	59.4 (397/668)	1.84 (0.98, 3.43)
Not known/missing	10.3 (4/39)	15.2 (107/704)	1.10 (0.29, 4.19)	15.4	(14/91/)	18.4 (123/668)	1.30 (0.59, 2.86)
Age at sexual debut							
Under 13 years of age	2.6 (1/39)	2.6 (18/704)	1.00	5.5	(5/91/)	5.8 (39/668)	1.00
13 years of age or over	92.3 (36/39)	91.8 (624/704)	0.96 (0.13, 7.42)	85.7	(78/91/)	78.6 (525/668)	0.86 (0.33, 2.26)
Not known/missing	5.1 (0/39)	5.6 (62/704)	0.56 (0.13, 2.38)	10.0	(8/91/)	15.6 (104/668)	0.52 (0.24, 1.10)
Partner age at sexual debut							
Same age or < 3 years older/younger	92.3 (36/39)	30.7 (216/704)	1.00	84.6	(77/91/)	54.2 (362/668)	1.00
3+ years older	**2.6 (1/39)**	**58.2 (410/704)**	**0.02 (0.00, 0.11)**	**5.5 (5/91/)**		**26.6 (178/668)**	**0.13 (0.05, 0.33)**
Not known/missing	**5.1 (2/39)**	**11.1 (78/704)**	**0.15 (0.04, 0.65)**	**9.9 (9/91/)**		**19.2 (128/668)**	**0.33 (0.16, 0.68)**
Felt pressured at sexual debut							
Yes	10.3 (4/39)	12.1 (85/704)	1.00	2.2 (2/91/)		12.6 (84/668)	1.00
No	84.6 (33/39)	71.6 (504/704)	1.39 (0.48, 4.03)	**89.0 (81/91/)**		**66.2 (442/668)**	**7.70 (1.86, 31.91)**
Not known/missing	5.1 (2/39)	16.3 (115/704)	0.37 (0.07, 2.07)	8.8 (8/91/)		21.3 (142/668)	2.37 (0.49, 11.40)
Sexual debut was under the influence of drugs or alcohol							
Yes	33.3 (13/39)	27.0 (190/704)	1.00	44.0 (40/91/)		30.2 (202/668)	1.00
No	66.7 (26/39)	67.6 (476/704)	1.25 (0.63, 2.49)	**50.5 (46/91/)**		**61.5 (411/668)**	**0.57 (0.36, 0.89)**
Not known/missing	0.0 (0/39)	5.4 (38/704)	n/a	5.5 (5/91/)		8.2 (55/668)	0.50 (0.17, 1.22)
Discussed using condoms with partner at sexual debut							
Yes	28.2 (11/39)	40.9 (288/704)	1.00	22.0 (20/91/)		39.7 (265/668)	1.00
No	**51.3 (20/39)**	**31.7 (223/704)**	**2.35 (1.10, 5.00)**	**50.5 (46/91/)**		**28.9 (193/668)**	**3.16 (1.81, 5.51)**
Not known/missing	20.5 (8/39)	27.4 (193/704)	1.09 (0.43, 2.75)	27.5 (25/91/)		31.4 (210/668)	1.58 (0.85, 2.92)
Has had more than the median number of partners							
1–2 sexual partners	30.8 (12/39)	54.9 (386/704)	1.00	23.1 (21/91/)		50.5 (337/668)	1.00
3+ sexual partners	**53.8 (21/39)**	**31.4 (221/704)**	**3.06 (1.48, 6.33)**	**59.3 (54/91/)**		**30.0 (201/668)**	**4.33 (2.54, 7.39)**
Not known/missing	15.4 (6/39)	13.7 (97704)	2.01 (0.74, 5.49)	**17.6 (16/91/)**		**19.5 (130/668)**	**1.98 (1.00, 3.91)**

Results significant at *p*<0.05 are in bold

Among sexually active AES and MES pupils, there was no significant differences in reported rates of unplanned sexual debut or the age at which sexual debut occurred (Table 5). AES pupils had reduced odds of reporting that their partner at sexual debut was 3 or more years older than they were (Male OR = 0.33, 95% CI 0.16–0.68; Female OR 0.02, 95% CI 0.00–0.11). Compared with MES pupils, AES pupils had: increased odds of reporting that they had not felt pressured at sexual debut (Males only OR = 7.70, 95% CI 1.86, 31.91); reduced odds of their sexual debut occurring under the influence of drugs and alcohol (Males only OR = 0.57, 95% CI 0.36, 0.89); and increased odds of not discussing using condoms prior to sexual debut (Male OR = 3.16, 95% CI 1.81, 5.51, Female OR = 2.35, 95% CI 1.10, 5.00). Finally, compared with MES pupils, AES pupils had increased odds of reporting that they had more than the median number (three or more) of sexual partners (Male OR = 4.33, 95% CI 2.54, 7.39, Female OR = 3.06, 95% CI 1.48, 6.33).

### RQ3: can potential differences in rates of PSU and sexual risk-taking between AES and MES pupils in the UK be explained by individual (sex, age and socioeconomic circumstances) and interpersonal (family, education, and peer) factors?

#### Poly-substance use

Compared with MES pupils, odds of PSU (using all three substances) at least once a week were increased for AES pupils, after adjustment for individual factors of age, sex and socioeconomic circumstances (Table 6: PSU Model 1, OR = 3.69, 95% CI 2.69, 5.06). Adjusting for family-focussed interpersonal factors, including frequency of parent-child arguments, parent-child connectedness, family time, spending money and parental monitoring reduced the odds of increased PSU (Model 2, OR = 3.32, 95% CI 2.36, 4.68), as did adjusting for interpersonal factors focussed on education, e.g. intended school leaving ages, expectations of education, employment and training, and expectations of parenthood (Model 3, OR = 2.46, 95% CI 1.71, 3.52). Adding interpersonal peer factors (Model 4) somewhat weakened the association between AES and PSU, however AES pupils remained significantly more likely to report weekly PSU than MES pupils (OR = 1.73, 95% CI 1.15, 2.60).

**Table 6:** Poly substance use amongst AES and MES pupils

	Poly substance use^a^									
	Model 1			Model 2			Model 3	Model 4		
	OR (95% CI)			OR (95% CI)			OR (95% CI)	OR (95% CI)		
School setting										
MES	1.00			1.00			1.00	1.00		
AES	**3.69 (2.69, 5.06)**			**3.32 (2.36, .68)**			**2.46 (1.71, 3.52)**	**1.73 (1.15, 2.60)**		
Age										
Older than 15.5 years	1.00			1.00			1.00	1.00		
Younger than 15.5 years	1.05 (0.88, 1.26)			1.03 (0.85, 1.23)			1.02 (0.85, 1.24)	1.0 (0.80, 1.21)		
Sex										
Male	1.00			1.00			1.00	1.00		
Female	**0.83 (0.70, 1.00)**			0.84 (0.69, 1.01)			0.89 (0.73, 1.07)	**0.69 (0.56, 0.86)**		
Family composition										
Lives with both biological parents	1.00			1.00			1.00	1.00		
Lives in single parent household	**1.83 (1.48, 2.27)**			1.70 (1.36, 2.12)			1.58 (1.26, 1.98)	**1.37 (1.07, 1.76)**		
Lives in a reconstituted/step family	**2.07 (1.55, 2.77)**			1.82 (1.35, 2.46)			1.72 (1.27, 2.34)	**1.41 (1.00, 1.97)**		
Lives in residential or foster care	**3.23 (2.01, 5.17)**			3.09 (1.90, 5.05)			2.60 (1.57, 4.28)	**2.13 (1.23, 3.71)**		
Paternal employment status										
Full time employment	1.00			1.00			1.00	1.00		
Part time employment	0.80 (0.50, 1.28)			0.71 (0.44, 1.16)			0.65 (0.40, 1.07)	0.6 (0.36, 1.27)		
Unemployed	0.95 (0.68, 1.33)			1.00 (0.71, 1.40)			0.93 (0.66, 1.32)	0.9 (0.60, 1.29)		
Maternal employment status										
Full time employment	1.00			1.00			1.00	1.00		
Part time employment	0.95 (0.76, 1.20)			0.98 (0.77, 1.24)			1.03 (0.81, 1.31)	1.06 (0.81, 1.38)		
Unemployed	0.95 (0.73, 1.24)			0.98 (0.75, 1.29)			0.98 (0.75, 1.30)	1.03 (0.76, 1.38)		
Frequency of parent-child arguments										
At least once a week				1.00			1.00	1.00		
Less than once a week				**0.45 (0.38, 0.58)**			**0.47 (0.35, 0.65)**	**0.58 (0.46, 0.72)**		
Parent-child connectedness										
High levels of reported connectedness				1.00			1.00	1.00		
Low levels of reported connectedness				1.15 (0.95, 1.40)			1.12 (0.92, 1.36)	1.20 (0.97, 1.49)		
Family time										
Frequently eats meals and go places as a family				1.00			1.00	1.00		
Infrequently eats meals and go places as a family				**1.86 (1.53, 2.26)**			**1.79 (1.46, 2.18)**	**1.42 (1.15, 1.77)**		
Spending money										
£24 or less per week				1.00			1.00	1.00		
£25 or more per week				**1.52 (1.24, 1.87)**			**1.41 (1.14, 1.74)**	**1.29 (1.02, 1.63)**		
Parental monitoring										
High levels of parental monitoring				1.00			1.00	1.00		
Low levels of parental monitoring				**1.49 (1.23, 1.80)**			**1.42 (1.17, 1.73)**	**1.50 (1.18, 1.80)**		
Intended school leaving age										
Remain at school beyond legal leaving age							1.00	1.00		
Leave school as soon as legally possible							**2.56 (2.06, 3.18)**	**1.52 (1.19, 1.94)**		
Expectations of employment, education and training										
Will be in education, employment or training							1.00	1.00		
Will not be in education, employment or training							0.73 (0.41, 1.31)	0.73 (0.39, 1.36)		
Expectations of parenthood										
Will be a parent							1.00	1.00		
Will not be a parent							0.91 (0.64, 1.28)	1.24 (0.85, 1.80)		
Peer behaviours										
Less than half of peers have left school								1.00		
More than half of peers have left school								1.14 (0.80, 1.62)		
Less than half of peers smoke tobacco								1.00		
More than half of peers smoke tobacco								**5.74 (4.52, 7.31)**		
Less than half of peers have had sex								1.00		
More than half of peers have had sex								**2.46 (1.96, 3.10)**		

^a^Missing/unknown categories had no associations with PSU, pre-consent age sexual activity or protected against pregnancy and STIs in all stages of analysis, so are omitted from this table

Results significant at *p*<0.05 are in bold

Looking beyond AES status, our fully adjusted model (Model 4) indicates that female pupils and pupils who reported that they had infrequent arguments with their parents had significantly lower odds of engaging in PSU. Factors that significantly elevated the odds of PSU included: living in a single parent or reconstituted household, living in residential and foster care, having greater amounts of spending money, spending less time with family, receiving low levels of parental monitoring, intending to leave school as soon as it was legally possible to do so, having peers who smoked and having peers who were sexually active.

#### Sexual risk behaviour: has had penetrative sex

Compared with MES pupils, odds of having had penetrative sex were increased for AES pupils, after adjusting for individual factors of age, sex and socioeconomic circumstances (Table 7, has had penetrative sex Model 1, OR = 2.89, 95% CI 2.08, 4.02). Adjusting for family-focussed interpersonal factors, marginally reduced the association between AES and having had penetrative sex (Model 2, OR = 2.46, 95% CI 1.74, 3.74). Further adjustment for interpersonal factors focussed on education (e.g. intended school leaving ages, expectations of education, employment and training, and expectations of parenthood) attenuated the increased risk of having had penetrative sex, suggesting that the increased risk observed for AES pupils can be explained by the combined influence of socio-economic circumstances, parent-child relationships, intended school leaving age and expectations towards education, employment, training and parenthood (Model 3, OR = 1.41, 95% CI 0.98, 2.04). Further attenuations were observed after adjusting for interpersonal peer factors (Model 4, OR = 0.99, 95% CI 0.65, 1.50).

**Table 7:** Sexual risk behaviours among AES and MES pupils

	Sexual activity^a^										Protected against pregnancy and STIs^a^							
	Model 1			Model 2			Model 3			Model 4	Model 1			Model 2			Model 3	Model 4
	OR (95% CI)			OR (95% CI)			OR (95% CI)			OR (95% CI)	OR (95% CI)			OR (95% CI)			OR (95% CI)	OR (95% CI)
School setting																		
MES	1.00			1.00			1.00			1.00	1.00			1.00			1.00	1.00
AES	**2.89 (2.08, 4.02)**			**2.46 (1.74, 3.47)**			1.41 (0.98, 2.04)			0.99 (0.65, 1.50)	**0.55 (0.37, 0.83)**			**0.57 (0.37, 0.86)**			0.74 (0.47, 1.15)	0.79 (0.50, 1.25)
Age																		
Older than 15.5 years	1.00			1.00			1.00			1.00	1.00			1.00			1.00	1.00
Younger than 15.5 years	**1.31 (1.15, 1.51)**			**1.28 (1.11, 1.48)**			1.26 (1.09, 1.46)			1.27 (1.09, 1.49)	0.84 (0.67, 1.04)			0.84 (0.67, 1.06)			0.86 (0.68, 1.08)	0.86 (0.69, 1.09)
Sex																		
Male	1.00			1.00			1.00			1.00	1.00			1.00			1.00	1.00
Female	**1.17 (1.02, 1.34)**			**1.20 (1.04, 1.39)**			1.34 (1.15, 1.55)			1.19 (1.02, 1.40)	0.92 (0.74, 1.15)			0.91 (0.72, 1.14)			0.85 (0.68, 1.07)	0.88 (0.70, 1.11)
Family composition																		
Lives with both biological parents	1.00			1.00			1.00			1.00	1.00			1.00			1.00	1.00
Lives in single parent household	**1.68 (1.42, 2.00)**			**1.61 (1.35, 1.92)**			**1.55 (1.29, 1.86)**			**1.38 (1.13, 1.68)**	1.11 (0.85, 1.45)			1.12 (0.86, 1.47)			1.18 (0.90, 1.55)	1.21 (0.92, 1.60)
Lives in a reconstituted/step family	**2.07 (1.63, 2.64)**			**1.89 (1.47, 2.41)**			**1.88 (1.46, 2.42)**			**1.58 (1.20, 2.09)**	0.92 (0.65, 1.32)			0.93 (0.65, 1.33)			0.97 (0.67, 1.40)	1.01 (0.70, 1.45)
Lives in residential or foster care	**2.17 (1.36, 3.47)**			**2.18 (1.34, 3.54)**			**1.80 (1.09, 3.00)**			**1.82 (1.06, 3.14)**	0.74 (0.40, 1.39)			0.75 (0.40, 1.41)			0.85 (0.45, 1.62)	0.89 (0.46, 1.70)
Paternal employment status																		
Full time employment	1.00			1.00			1.00			1.00	1.00			1.00			1.00	1.00
Part time employment	0.83 (0.58, 1.18)			0.78 (0.54, 1.12)			0.69 (0.47, 1.00)			0.68 (0.45, 1.03)	0.97 (0.55, 1.71)			0.99 (0.55, 1.17)			0.83 (0.57, 1.21)	0.85 (0.58, 1.24)
Unemployed	1.05 (0.80, 1.36)			1.09 (0.83, 1.43)			0.99 (0.75, 1.31)			1.02 (0.75, 1.37)	1.12 (0.74, 1.68)			1.06 (0.70, 1.60)			1.13 (0.74, 1.71)	1.13 (0.74, 1.72)
Maternal employment status																		
Full time employment	1.00			1.00			1.00			1.00	1.00			1.00			1.00	1.00
Part time employment	**0.73 (0.62, 0.87)**			**0.74 (0.62, 0.89)**			**0.78 (0.65, 0.94)**			**0.80 (0.66, 0.97)**	0.89 (0.67, 1.20)			0.90 (0.67, 1.20)			0.88 (0.65, 1.18)	0.89 (0.66, 1.21)
Unemployed	**0.75 (0.61, 0.92)**			**0.77 (0.62, 0.95)**			**0.77 (0.62, 0.96)**			**0.77 (0.61, 0.97)**	0.65 (0.47, 0.90)			0.65 (0.47, 0.90)			0.65 (0.47, 0.91)	0.65 (0.46, 0.90)
Frequency of parent-child arguments																		
At least once a week				1.00			1.00			1.00				1.00			1.00	1.00
Less than once a week				**0.58 (0.50, 0.67)**			**0.61 (0.53, 0.72)**			**0.72 (0.61, 0.84)**				1.17 (0.92, 1.49)			1.13 (0.88, 1.44)	1.10 (0.86, 1.41)
Parent-child connectedness																		
High levels of reported connectedness				1.00			1.00			1.00				1.00			1.00	1.00
Low levels of reported connectedness				0.95 (0.82, 1.10)			0.89 (0.76, 1.04)			0.87 (0.74, 1.03)				0.93 (0.73, 1.18)			0.96 (0.76, 1.22)	0.95 (0.75, 1.21)
Family time																		
Frequently eats meals and go places as a family				1.00			1.00			1.00				1.00			1.00	1.00
Infrequently eats meals and go places as a family				**1.55 (1.34, 1.79)**			**1.53 (1.32, 1.77)**			**1.33 (1.13, 1.57)**				0.92 (0.72, 1.16)			0.91 (0.72, 1.16)	0.96 (0.75, 1.22)
Spending money																		
£24 or less per week				1.00			1.00			1.00				1.00			1.00	1.00
£25 or more per week				**1.74 (1.48, 2.06)**			**1.64 (1.39, 1.95)**			**1.55 (1.29, 1.87)**				0.88 (0.69, 1.13)			0.91 (0.71, 1.17)	0.93 (0.72, 1.20)
Parental monitoring																		
High levels of parental monitoring				1.00			1.00			1.00				1.00			1.00	1.00
Low levels of parental monitoring				**1.32 (1.14, 1.53)**			**1.24 (1.06, 1.44)**			**1.21 (1.02, 1.42)**				0.92 (0.73, 1.16)			0.95 (0.75, 1.20)	0.97 (0.76, 1.23)
Intended school leaving age																		
Remain at school beyond legal leaving age							1.00			1.00							1.00	1.00
Leave school as soon as legally possible							**2.85 (2.36, 3.44)**			**1.89 (1.54, 2.33)**							**0.63 (0.49, 0.81)**	**0.70 (0.54, 0.91)**
Expectations of employment, education and training																		
Will be in education, employment or training							1.00			1.00							1.00	1.00
Will not be in education, employment or training							0.81 (0.52, 1.27)			0.79 (0.49, 1.29)							0.65 (0.33, 1.30)	0.65 (0.33, 1.29)
Expectations of parenthood																		
Will be a parent							1.00			1.00							1.00	1.00
Will not be a parent							**0.36 (0.27, 0.50)**			**0.42 (0.30, 0.59)**							**1.58 (1.10, 2.30)**	**1.58 (1.09, 2.30)**
Peer behaviours																		
Less than half of peers have left school										1.00								1.00
More than half of peers have left school										**5.10 (4.19, 6.19)**								1.01 (0.69, 1.46)
Less than half of peers smoke tobacco										1.00								1.00
More than half of peers smoke tobacco										**2.01 (1.55, 2.61)**								**0.74 (0.56, 0.98)**
Less than half of peers have had sex										1.00								1.00
More than half of peers have had sex										**1.89 (1.21, 2.96)**								**0.77 (0.60, 0.99)**

^a^Missing/unknown categories had no associations with PSU, pre-consent age sexual activity or protected against pregnancy and STIs in all stages of analysis, so are omitted from this table. The only exception to this was for the relationship between parenting aspirations and pre-consent sexual activity, where a significant effect was seen in Model 3 (OR = 0.46, 95% CI 0.33, 0.64) and Model 4 (OR = 0.50, 95% CI 0.35, 0.72) suggesting that after controlling for all other factors, pupils who provided missing data to this question or were not definitively certain about their intentions towards parenthood were more likely to engage in pre-consent sexual activity and therefore had an elevated risk of early parenthood

Results significant at *p*<0.05 are in bold

Looking beyond AES status, our fully adjusted model (Model 4) indicates that pupils who reported that they did not expect to become parents in the next three years had significantly lowered odds of being sexually active. Other protective factors included having a mother who was not in full time employment and reporting infrequent arguments with parents. Factors that significantly increased the odds of young people in our sample being sexually active included: living in a single parent or reconstituted household, living in residential and foster care, having greater amounts of spending money, spending less time with family, receiving low levels of parental monitoring, intending to leave school as soon as it was legally possible to do so, having peers who had already left education, having peers who smoked and having peers who were sexually active.

#### Sexual risk behaviour: protection against pregnancy and STIs

Compared with MES pupils, AES pupils had reduced odds of having been protected against both pregnancy and STIs (either through abstinence or by using condoms or condoms plus another contraceptive at sexual debut or beyond), after adjusting for individual factors of age, sex and socioeconomic circumstances (Table 7, was protected against pregnancy and STIs Model 1, OR = 0.55, 95% CI 0.37, 0.83). Adjustment for family-focussed interpersonal factors, marginally accentuated this finding (Model 2, OR = 0.57, 95% CI 0.37, 0.86). Further adjustment for interpersonal factors focussed on education (e.g. intended school leaving ages, expectations of education, employment and training, and expectations of parenthood) fully attenuated the reduced odds of being protected against pregnancy and STIs observed among AES pupils, suggesting that the combined influence of socio-economic circumstances, parent-child relationships, intended school leaving age and expectations towards education, employment, training and parenthood fully explain differences in abstinence and condom use between AES and MES pupils (Model 3, OR = 0.74, 95% CI 0.47, 1.15). No further attenuation was observed after adjusting for interpersonal peer factors (Model 4, OR = 0.79, 95% CI 0.50, 1.25).

Looking beyond AES status, our fully adjusted model (Model 4) indicates that pupils who reported that they did not expect to become parents in the next three years had significantly increased odds of having always been protected against both pregnancy and STIs. Factors that significantly reduced the odds of pupils consistently protecting themselves against pregnancy and STIs included: intending to leave education as soon as it was legally possible to do so, having peers who smoked and having peers who were sexually active.

## Discussion

In order to better understand heath inequalities in the UK this paper drew upon the social ecological model to compare the levels of health risk behaviours exhibited by AES pupils [13] and explore the contexts in which these behaviours occurred [63]. Our results show that AES pupils are consistently more disadvantaged than their contemporaries in MES, and are more likely to: grow up in under-employed households; experience family breakdown; be looked after in residential or foster care and have limited educational aspirations (RQ1). Looking specifically at health risk behaviours, our results demonstrate that, compared with MES pupils, AES pupils have higher rates of PSU and more frequently engage in sexual risk behaviours, including having had penetrative sex, failure to consistently protect against pregnancy and STIs, increased numbers of sexual partners and having sex under the influence of drugs and alcohol (RQ2). These findings are consistent with the existing international literature identifying AES pupils as a vulnerable population of young people [16, 51–54] who exhibit high levels of health risk behaviours [34, 36, 44].

The absolute rates of health risk behaviours that were seen among our sample are lower than those reported in the USA [34] and New Zealand [36]. Differences in sample characteristics may help to explain this finding. Our sample of AES pupils consisted of pupils who were excluded from MES as a result of SEN and/or SEBD. In contrast, the data that was generated from the USA focussed upon pupils in schools which served as alternatives to expulsion or imprisonment, or provided remediation or rehabilitation for pupils with SEBD [34], while the data from New Zealand focussed specifically upon young people with substantial histories of school refusal or exclusion [36]. It is possible that including SEN pupils within our sample of AES may have diluted the levels of risk taking observed as it is known that while SEN pupils have significantly elevated levels of health risk behaviours compared to MES, the greatest levels of risk are seen for SEBD pupils [14]. However, while we report lower levels of risk among our AES sample, it is important to note that the same pattern of elevated risk is present for AES when compared with MES.

There have been a number of studies which have identified that AES pupils exhibit significantly higher levels of health risk behaviours than MES pupils, however, to our knowledge there have been no studies that have focussed upon identifying why AES pupils are significantly more likely than their contemporaries in MES to use substances and engage in earlier and risky sex [50]. In this study we used multivariable analyses to show for the first time that increased rates of sexual activity and failure to consistently protect against pregnancy and STIs among AES pupils can be fully explained by the combined effects of socioeconomic, familial and educational disadvantage among AES pupils (RQ3). These findings are in line with the extant literature showing that deprivation, living in a lone-parent household, having low levels of parental monitoring, living in a care/foster home and having limited educational/career aspirations are all significantly and independently associated with earlier and riskier sexual behaviour among adolescents [25, 26]. However, where our findings add to the evidence base is by identifying that it is the accumulation of these risk factors that underscores the inequalities in sexual health outcomes observed between AES and MES pupils.

Whereas our results demonstrate that increased levels of sexual risk taking in AES versus MES pupils can be explained by the greater levels of disadvantage among AES pupils, our results demonstrate that the increased risk of PSU in AES versus MES pupils cannot be fully explained by differences in the socio-economic, familial, educational and peer backgrounds of these pupils (RQ3). That increased rates of PSU among AES pupils were only partially explained by the explanatory variables investigated suggests that PSU may be attributable to other unmeasured factors. Evidence from the international literature suggests that substance use amongst AES pupils is associated with mental health difficulties, juvenile offending and childhood abuse histories [37–39, 60, 77], which were not investigated in our study. For instance, it has been demonstrated that among AES pupils: inhalant use is associated with depression and carrying a weapon during adolescence [37]; alcohol use is associated with a history of coercive sexual experiences [39]; and cannabis, cocaine and codeine use by young women is associated with a history of being sexually abused [77]. Further research should investigate the effects of these socio-environmental factors on AES pupils’ health risk behaviours in order to help feed in to future intervention development. School-effects research is also needed to understand how organisational factors such as learning cultures and peer mix within AES settings may serve to protect against or accentuate exposure to health risk behaviours.

### Study limitations and implications for future research

A major strength of this exploratory study is that the methodology was the same for AES and MES schools, with data collected from the same regional areas and at the same time point. This makes our comparison of vulnerable adolescents in AES with MES pupils more valid than drawing upon data collected using disparate measures collected at differing time points. Despite this, six limitations to our analysis must be acknowledged. First, the small cross-sectional sample size and low numbers per setting meant that separate multivariate analyses for gender were not undertaken and instead gender was adjusted for in the results. It also means that we are not able to explore the causal pathways that underscore adoption of risk behaviours. For instance, whilst we know from our data that AES pupils had higher levels of exposure to risky peers we do not know whether this exposure pre-dated or followed on from the adoption of health risk behaviours. To explore this in more depth, prospective data is needed. Second, analyses did not account for clustering of pupils by school due to the small number of AES pupils within each school (n_min_ = 5, n_max_ = 36). Thus, we did not adjust for the likelihood that school level effects would be seen within the data. It is therefore possible that the standard errors were underestimated, and hence the significance levels overemphasised, meaning that we were more likely to observe significant effects. A larger sample for AES would counter many of these issues and allow better determination of the strength of associations.

A third limitation is the potential for recall bias among pupil responses. A number of studies have assessed the reliability and validity of recall information relating to alcohol, tobacco and drug use, concluding that in longitudinal studies the recalled mean age of first engaging in substance use and sexual risk behaviours increases as individuals age [78–80]. For sexual initiation it has been shown that recall biases are most pronounced among individuals who engage in earlier risk taking, suggesting a tendency to conceal experiences that they later to perceive to have been contrary to social norms [80]. In order to minimise recall bias for the reporting of substances, we chose not to ask pupils about the age at which they first used substances and instead asked about the use of alcohol, tobacco and cannabis within the thirty days prior to being surveyed. For sexual experiences, however, we did ask pupils to recall the age at which their sexual debut occurred.

How we asked pupils about their sexual risk behaviours may have affected our results, and indeed, one of the findings that was contrary to what we would have expected was the lack of reported difference between AES and MES pupils in the prevalence of sexual activity under the age of 13. While it is possible that this lack of difference is due to recall bias, sexual activity under the age of 13 is relatively rare, and the percentages that we have reported are comparable to those seen in national surveillance programmes [81]. We also know from previous research evaluating the impact of sexual health and relationships education that there is little difference in adolescent sexual health outcomes as measured by self-report and objective health surveillance data within Scotland [69]. Thus, while we cannot be certain that our results do not reflect over- or under-reporting of behaviours, we are reassured that they are consistent with the wider evidence base.

The fourth limitation of our study is that we do not have sufficient information about the reasons why pupils attended AES to explore how health risk behaviours patterned by pupil type. As highlighted earlier, the rates of risk taking behaviour in our sample are lower than those observed in the USA and New Zealand [34, 36]. This may be explained by our inclusion of AES pupils with both SEN and SEBD histories in our sample. Data collected in the Netherlands shows that whilst SEN pupils have significantly elevated levels of health risk behaviours compared to MES, the greatest levels of risk are seen for SEBD pupils [14]. That differential effects have been observed elsewhere is important as without being able to disaggregate pupil type we cannot say whether our results are over- or under-representative of the scale of health risk behaviours among AES. Additional UK-based research is therefore needed to explore health inequalities within AES settings. Furthermore, as it can be hypothesised that some children in AES may have less opportunity to be exposed to risky peers — for instance, pupils with autistic spectrum disorders who have difficulty forming social relationships, or pupils with complex health needs who may receive higher levels of monitoring by parents and teachers — this research should focus on furthering our understandings of the potential protective mechanisms for AES pupils.

Fifth, we are aware that our study did not capture the behaviours of all young people in MES, as those absent due to truancy, school refusal and short- or long-term health needs were not surveyed. Not including these individuals in our analysis may potentially have diluted risk taking levels among MES pupils. Similarly, by not capturing the behaviours of those individuals who meet the UK Department of Education’s definition of AES but are home schooled we may have under- or over-estimated risk among pupils with additional educational support need [82]. However, whilst these limitations exist, the magnitude of the effects observed suggest that significant differences do exist between the two settings. Thus, further research that seeks to understand why this occurs would be beneficial. Finally, while our analysis drew upon principles of the social ecological model to explore how individual and interpersonal factors could be used to explore inequalities in health risk behaviours between AES and MES pupils, we did not consider how organisational-, community- and policy-related factors were associated with health risk behaviours among AES pupils [64]. While future research is needed to explore the role that these factors can play in accentuating or mitigating health risk behaviours among AES pupils, the existing literature base suggests that interventions designed to promote social inclusion and connectedness at the organisational- and community-level can reduce the risk of engaging in health risk behaviours among adolescents [83, 84].

### Implications for policy and practice

AES pupils may not be easily targeted by typical population level public health programmes. Our findings highlight that the inequalities in sexual health risk behaviours observed between AES and MES pupils are attributable not to AES, but instead to differences in background variables, particularly socio-economic, familial and educational engagement and expectations. Educational policy makers should be cognisant that while AES pupils are outside mainstream education, they are still accessible through the education system. This means that AES are uniquely placed to both help raise young people’s aspirations and implement harm minimisation programmes to reduce health risk behaviours. Given the relationship between substance use and sexual risk taking behaviour, these programmes should include discussions of the relationship between drug and alcohol use, and sexual risk behaviour [74].

Specially designed health education packages for AES which acknowledge the clustering of health risk behaviours with shared underlying determinants may be beneficial in targeting this vulnerable population [24]. At the organisational-level, potential interventions may include whole school interventions aimed at changing school culture and ethos [83, 84], or multi-setting interventions that combine school-based education programmes with activities designed to strengthen the relationships between schools, parents and communities [85, 86]. For instance, the Gatehouse Project used a standardised intervention process within schools that incorporated feeding back the results of pupil surveys on the social climate and safety of schools to school-based action teams consisting of management, teaching and auxiliary staff in order to enable them to work with external facilitators to develop action plans and policies focussed upon promoting whole-school change, and select and implement health promotion tools that reflected the needs of pupils within the school [87]. This approach, which focussed upon improving social inclusion and connectedness within the school setting, was associated with a significant reduction in early initiation of sexual intercourse and risky behaviour (measured using a composite variable of substance use, antisocial behaviour and sexual intercourse) four years after implementation [84].

Due to our findings that AES pupils are more likely than their peers in MES to experience family breakdown and be exposed to risky peers, positive youth development programmes could also be used to reduce health risk behaviours. These programmes operate at the interpersonal level by providing mentoring and befriending to build relationship skills, promote self-confidence and self-efficacy, challenge social norms that promote engagement in risk-taking behaviour and increase educational aspirations by providing access to positive adult role models [88]. A systematic review of experimental and quasi-experimental evaluations of Positive Youth Development programmes in the USA identified 15 programmes that had resulted in increased condom usage, reduced frequency of sexual intercourse, reduced numbers of sexual partners and reduced levels of teenage pregnancy. The authors concluded that PDY programmes that were effective at reducing sexual risk taking specifically focussed upon “promoting prosocial bonding, cognitive competence, social competence, emotional competence, belief in the future, and self-determination” [89]. Given the increased propensity of AES pupils to have socio-emotional difficulties and engage in antisocial behaviours [16–18] future research is needed to explore whether such interventions would reduce health risk behaviours in this population.

Finally it should be recognised that AES pupils are at greater risk of experiencing early parenthood, and indeed, our results show that early parenthood was something that around a fifth of AES pupils envisioned for themselves. While for some young people, early parenthood can be a positive experience [90–92] it is known that early parenthood can negatively affect the socio-economic and health status of parent and child and result in young people being further excluded from the labour market; both of which can further increase social exclusion [93]. Thus, while interventions designed to address health risk behaviours among AES are needed, these should be balanced by exploring the benefits of interventions that also have the potential to reduce intergenerational transmission of social exclusion. Promising examples include the Family Nurse Partnership which works with young parents to improve parent-child attachment while providing access to health, social and welfare services [94] and the Seattle Social Development Project designed for primary school children which aims to improve pupils’ commitment to school and parent-child bonding [95]. These programmes, which use an early intervention model, are focussed upon improving health outcomes for children, and may be particularly advantageous for use with young parents as they have been shown to have long-term effects upon the sexual risk behaviours of offspring.

## Conclusion

AES pupils are a particularly vulnerable group, and this paper provides the first UK evidence of the substantial health gap between AES and MES pupils. For sexual risk, differences between AES and MES pupils can be explained by the combined effects of socioeconomic, familial and educational disadvantage experienced by AES pupils. Thus, evidence-based interventions to improve the sexual health of AES, especially those related to raising educational aspiration and minimising intentions towards early pregnancy and parenthood, may be particularly beneficial among AES. As an increased risk for PSU remained for AES vs MES pupils after adjusting for these factors and the influence of peer behaviours, further research is needed to explore additional factors that may help attenuate PSU, such as attitudes to risk, mental health difficulties, juvenile offending and abuse histories.

Our findings highlight the need for bespoke intervention development and feasibility work to learn how best to support pupils in AES to reduce health risk behaviours. Beyond this, policy makers should be aware of and act on the evidence that broader social changes are required to reduce the marginalisation and social exclusion of young people excluded from MES.

## Abbreviations

AES: Alternative education settings
MES: Mainstream education schools
PSU: Poly-substance use
STIs: Sexually transmitted infections
